# Next-Generation Sequencing Based Gut Resistome Profiling of Broiler Chickens Infected with Multidrug-Resistant *Escherichia coli*

**DOI:** 10.3390/ani10122350

**Published:** 2020-12-09

**Authors:** Ome Kalsoom Afridi, Johar Ali, Jeong Ho Chang

**Affiliations:** 1 Department of Biology Education, Kyungpook National University, 80 Daehak-ro, Buk-gu, Daegu 41566, Korea; ummeafridi@gmail.com; 2 Center for Genome Sciences, Rehman Medical College, Hayatabad, Peshawar, Khyber Pakhtunkhwa 25000, Pakistan; 3 Executive Development Center, Sukkur Institute of Business Administration University, Sindh 65200, Pakistan

**Keywords:** broiler chickens, fecal microbiota, shotgun metagenome sequencing, dysbiosis, antibiotic resistance genes, multidrug resistance

## Abstract

**Simple Summary:**

Antimicrobial resistance acquired an endemic status in the Pakistan poultry sector. A cross-sectional study was designed to investigate the fecal microbiome and resistome of broiler chickens infected with multidrug-resistant *Escherichia coli* using next-generation sequencing. Results show the widespread presence of diverse antibiotic resistance genes, virulence-associated genes, plasmid replicon types, and dysbiotic fecal microbial communities. Results indicate that antibiotic resistance altered the fecal microbial community structure of broiler chickens. The use of next-generation sequencing in this study documents a robust and cost-effective approach to study the fecal microbiome and resistome diversities of broiler chickens.

**Abstract:**

The study was designed to investigate the fecal microbiome and resistome of broiler chickens infected with multidrug-resistant (MDR) *Escherichia coli* (*E. coli*). Fecal samples (*n* = 410) from broiler chickens were collected from thirteen randomly selected sites of Khyber Pakhtunkhwa and screened for the presence of MDR *E. coli*. Upon initial screening, thirteen (13) MDR *E. coli* isolates were then subjected to shotgun metagenome next-generation sequencing (NGS). NGS based resistome analysis identified the multidrug efflux pump system-related genes at the highest prevalence (36%) followed by aminoglycoside (26.1%), tetracycline (15.9%), macrolide-lincosamide-streptogramin (9.6%), beta-lactam (6.6%), rifampin (2%), sulphonamide (1.3%), phenicol (0.91%), vancomycin (0.62%), trimethoprim (0.34%), colistin (0.30%), and quinolone (0.33%). The most abundant virulence-associated genes (VAGs) identified were *iro*N, *iut*A, *iss*, and *iuc*A. NGS based taxonomic profiling at the phylum level revealed the predominance of Proteobacteria (38.9%) followed by Firmicutes (36.4%), Bacteroidetes (15.8%), and Tenericutes (8.9%). Furthermore, pathobionts such as *E. coli*, *Salmonella enterica*, *Klebsiella pneumoniae*, and *Shigella flexneri* belonging to the family Enterobacteriaceae were predominantly found. This study revealed the widespread presence of MDR genes, diverse VAGs, and a dysbiotic gut in the broiler chickens infected with MDR *E. coli* of Khyber Pakhtunkhwa for the first time using NGS.

## 1. Introduction

Antibiotic-resistant (AR) bacteria, particularly multidrug-resistant (MDR) bacteria, which emerged as deadly microbes, pose serious health issues to human and food animals across the globe [1]. It has been estimated that AR bacteria can be the cause of death of 10 million people each year across the globe [2]. Globally, antimicrobial resistance (AMR) has attained an alarming status due to the appearance of MDR and extensively drug-resistant (XDR) microbes [3]. Among the various AR and MDR microbes, *Escherichia coli* (*E. coli*) has been reported to be the most common bacteria causing serious economic losses in both human and veterinary medicine [4]. *E. coli*, being a common gut commensal, mostly acquires AMR horizontally, leading to several serious infections in both human and food borne animals [3,5]. Globally, poultry is considered as the largest sector of the food-producing industry [6]. In addition, poultry is considered to be fed the largest amount of antibiotics and to be an important producer of animal waste, thus significantly affecting the environment and contributing to the emergence of AR and MDR bacteria [7,8].

The use of various antimicrobials as growth promoters in veterinary medicine has been regulated in developed countries, but in Pakistan, antibiotics are still widely administered for therapeutic purposes and growth promotion in poultry [9,10]. A recent study suggested the extensive annual administration up to 568 tons of various antimicrobials in the poultry industry of Pakistan [10]. This widespread usage of antimicrobials in the Pakistan poultry sector increased the prevalence of MDR and XDR microbes, particularly *E. coli* [11]. The various antimicrobials used in poultry feed have been reported to profoundly affect the gut microbial community structure [12]. Understanding the effect of various antibiotics used commonly in the poultry feed on the gut microbiota is important as it can help in the targeted mitigation of various microbes [7]. Moreover, identifying the problematic strains associated with AMR can also help in designing AR mitigation strategies. A team of researchers identified a large antibiotic resistance genes (ARGs) pool to be linked to foodborne microbiota in various instant foods. The identified foodborne ARG pool was found to be related to various fermented milk products. This discovery helped in the identification of the various problematic probiotic strains which were then eventually removed from the product lines using a targeted mitigation strategy [13,14,15,16]. In Pakistan, culture-based antibiotic susceptibility tests (ASTs) are mostly practiced for AMR assessment. Culture-based AST has been reported to be imprecise, laborious, and time-consuming [17]. Therefore, robust methods such as next-generation sequencing (NGS) are warranted to expedite the AMR assessment. However, due to the high cost, NGS based methods are not commonly practiced in a developing country like Pakistan for AMR assessment. Shallow shotgun metagenome sequencing (SSMS) has been recommended by a recent study as a cost-effective alternative to the other NGS based methods [18]. Previous studies in Pakistan mostly focused on exploring the phenotypic AMR pattern, phylogenetic analysis, and abundance of ARGs in poultry [11,19,20]; however, the data related to chicken gut microbiota are scarce. Antibiotics commonly used in animal feed for both therapeutic and growth promotion, have been reported to alter the gut microbial community structure of food animals. However, data indicating the effect of antibiotics or AMR over the poultry gut microbiota are scarce in the Khyber Pakhtunkhwa region of Pakistan. To the best of our knowledge, we for the first time designed a cross-sectional study to ascertain the effect of antibiotics (excessively used in poultry farms for both therapeutic and growth promotion purposes) and MDR *E. coli* infection on the fecal microbiota and resistome of broiler chickens. Using a high throughput sequencing approach such as NGS, the present study aimed to characterize the fecal microbiome and resistome of broiler chickens infected with MDR *E. coli.*

## 2. Materials and Methods

### 2.1. Sampling

Poultry fecal samples (*n* = 410; directly from the cloaca) were collected from different poultry flocks located at various sites (*n* = 13) of Khyber Pakhtunkhwa, Pakistan. Information related to the commonly used antibiotics for growth promotion and therapeutic purposes has been collected from the farmers of selected poultry farms (Table S1). Fecal samples were streaked onto the 5% sheep blood agar and MacConkey agar media (Oxoid, Basingstoke, Hampshire, England) and incubated at 37 °C for 18 h. Sub-culturing was carried out using selective differential media eosin methylene blue agar [21]. The mature colonies were recognized as *E. coli* by using standard morphological (Gram stain, oxidase tests) and biochemical tests including methyl red, indole, Voges–Proskauer, citrate tests, and motility tests [22]. AST was performed using the Kirby–Bauer disc diffusion method. *E. coli* isolates were tested against aminoglycosides, neomycin, gentamycin, streptomycin, chloramphenicol, quinolones and fluoroquinolones, ofloxacin, nalidixic acid, sulfonamides, sulfamethoxazole, tetracycline, beta-lactam, ampicillin, nitrofurans, and cephalosporins as per Clinical and Laboratory Standards Institute (CLSI) guidelines [23].

### 2.2. Extraction of Genomic DNA

The PureLink^TM^ Microbiome DNA Purification Kit (Cat. no. A29790) was used to extract genomic DNA from the fecal samples (0.2 g) following the standard protocol (Thermo Fisher Scientific, Waltham, MA, USA). Qubit™ fluorometer was used to measure the concentration of genomic DNA following the manufacturer’s instructions (Invitrogen, Carlsbad, CA, USA) [24].

### 2.3. Preparation of DNA Sequencing Libraries

Sequencing libraries were prepared using the Illumina^®^ Nextera XT DNA Library Preparation Kit (FC-131-1096) and Nextera XT Index Kit v2 Set A (FC-131-2001) as per the manufacturer’s instructions (Illumina, San Diego, CA, USA) [25]. The normalized and pooled DNA libraries were loaded onto the flow cell for sequencing. Sequencing (2 × 150 bp paired-ends) was performed using the Illumina 300 cycles V2 MiSeq reagent kit (MS-103-1002, Illumina Inc., San Diego, CA, USA).

### 2.4. NGS Bioinformatics Analysis

Paired-end sequenced FASTQ files were analyzed using various bioinformatics software packages that are available publicly. De-multiplexing of all FASTQ data was performed using the CASAVA v1.8.2 package [26]. Trimmomatic v.0.36 software was used to remove technical biases, low-quality reads, and adapters [27]. Host DNA was removed from all sequenced samples using a computational tool KneadData v. 0.6.1. All the filtered FASTQ files were processed for taxonomic profiling using the MetaPhlAn 3.0 pipeline [28]. By using ARIBA v. 2.14.4 software (MEGAres database), all the filtered reads were subjected to the resistome analysis [29].

## 3. Results

### 3.1. Screening of Samples

A total of 59% (*n* = 242) of the screened samples were found to be *E. coli* positive. The percentage prevalence of *E. coli* positive samples (59%) differs among the 13 different poultry flocks. Poultry flock samples collected from Chargano Chowk Peshawar were found to be the most abundantly infected with *E. coli* (*n* = 30, 12.4%), followed by *Malakand (n* = 24, 9.9%), Charsadda (*n* = 23, 9.5%), and Nowshera (*n* = 20, 8.3%). The percentage abundance of *E. coli* positive samples among the different poultry flocks are given in Table S2. Overall, 94.6% (*n* = 229) of *E. coli* infected samples showed resistance to a single class of antibiotics while 5.3% (*n* = 13) of *E. coli* infected samples exhibited resistance to almost all the antibiotics and were labeled as MDR samples. Since our goal was to sequence the MDR *E. coli* samples, we therefore selected these 13 samples (one from each of the poultry flock) for subsequent Illumina high throughput sequencing.

### 3.2. Shotgun Metagenome Sequencing

We performed SSMS (0.127 million reads each) for taxonomic and resistome profiling. The NGS quality filter discarded reads with quality scores <30 and read lengths of less than 60 nucleotides. In total 1,653,070 filtered paired-end reads were obtained. The high-quality NGS reads (1,653,070) were processed for taxonomic and resistome analysis.

### 3.3. Resistome Analysis

**Figure 1:**
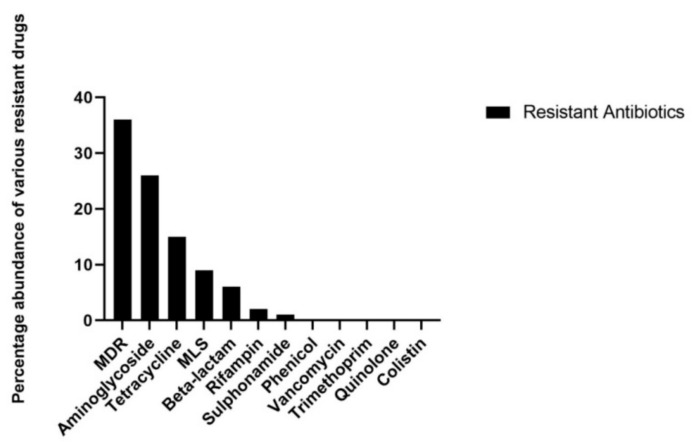
Antimicrobial resistance pattern of poultry fecal microbiota to various drugs. Multidrug resistance and macrolide-lincosamide-streptogramin are abbreviated as MDR and MLS, respectively.

A variety of ARGs conferring resistance to various antibiotics such as the multidrug efflux pump system, aminoglycoside, tetracycline, macrolide-lincosamide-streptogramin (MLS), beta-lactam, rifampin, sulphonamide, phenicol, vancomycin, trimethoprim, colistin, and quinolone were identified in all contigs datasets (Figure 1). NGS based resistome analysis revealed the predominance of ARGs to be 36% multidrug resistance (*n* = 70), 26.1% aminoglycoside (*n* = 20), 15.9% tetracycline (*n* = 15), 9.6% macrolide-lincosamide-streptogramin (*n* = 19), 6.6% beta-lactam (27), 2% rifampin (*n* = 2), 1.3% sulphonamide (*n* = 3), 0.91% phenicol (*n* = 2), 0.62% vancomycin (*n* = 1), 0.34% trimethoprim (*n* = 1), 0.30% colistin (*n* = 1), and 0.33% quinolone (*n* = 1) (Table 1).

### 3.4. Taxonomic Profiling Using NGS

**Figure 2:**
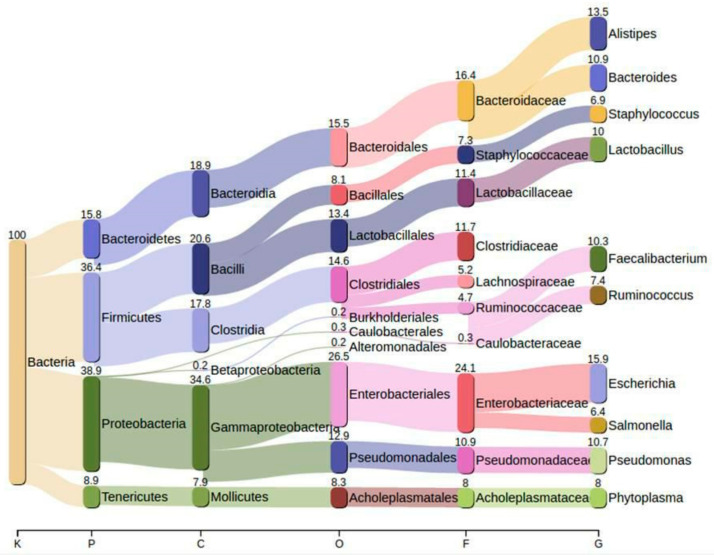
Taxonomic profiling of poultry fecal microbiota at various levels using Pavian derived from next-generation shotgun metagenomics. Different colored sidebars in the Sankey diagram show the relative abundance of bacterial communities in broiler chickens at various taxonomic levels including kingdom (K), phylum (P), class (C), order (O), family (F), and genus (G).

**Figure 3:**
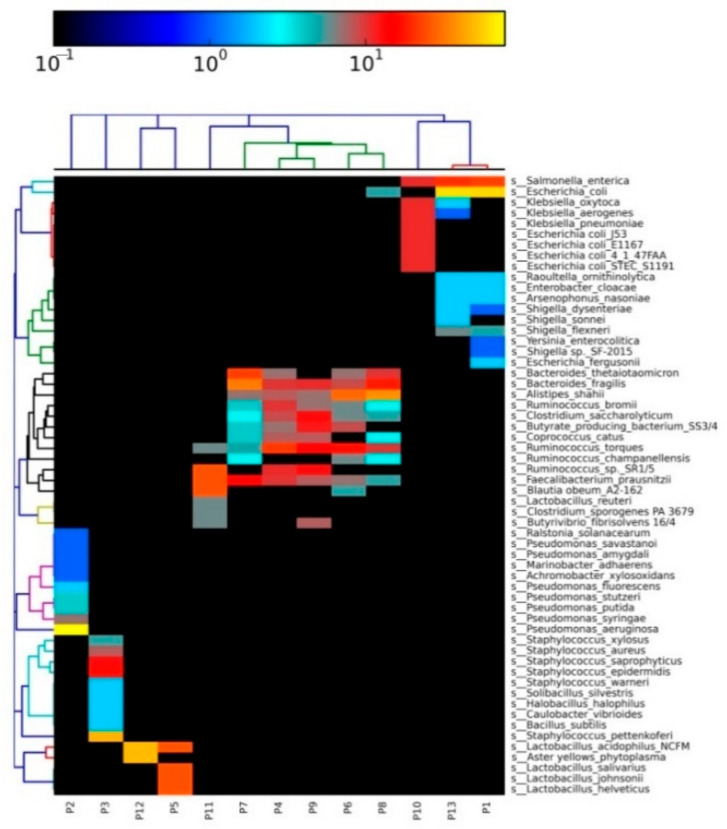
Heatmap showing the fecal microbiome profiling at the species level. All the bacterial species identified in the poultry fecal microbiome are hierarchically clustered using the Bray–Curtis dissimilarity matrix. P1 to P13 represents the sequenced shotgun metagenomic poultry samples (*n* = 13). The relative abundance of various bacterial species is indicated in logarithmic values (base 10).

Poultry fecal microbiome was composed of five bacterial phyla, namely Proteobacteria, Firmicutes, Bacteroidetes, and Tenericutes. Proteobacteria (38.9%) was identified as the most abundant phylum followed by Firmicutes (36.4%), Bacteroidetes (15.8%), and Tenericutes (8.9%). Enterobacteriaceae (24.1%) was identified to be the most dominant family among all the bacterial families (*n* = 10). Genus level microbial resolution revealed the predominance of *Escherichia* (15.9%) among all identified genera (*n* = 10). Bacterial profiling at various taxonomic levels is shown in Figure 2. Species-level profiling revealed the abundance of *E. coli*, *Salmonella enterica*, *Klebsiella pneumoniae*, and *Shigella flexneri* (Figure 3).

### 3.5. Virulence Genes

**Figure 4:**
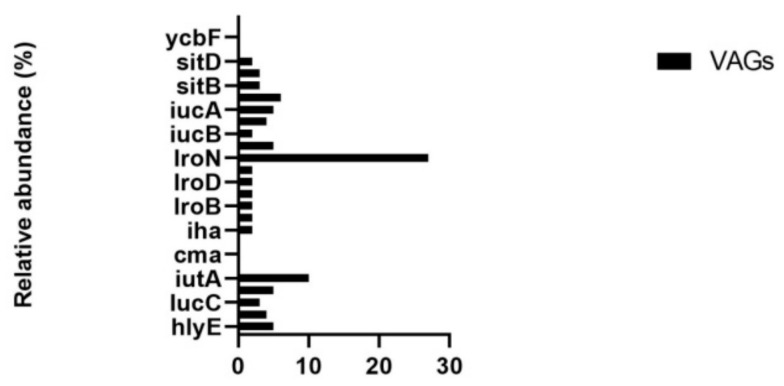
The relative abundance of various virulence-associated genes (VAGS).

A total of 26 virulence-associated genes (VAGs) were identified in all the metagenomic data sets. Most abundant VAGs were *IroN* (23%), *iutA* (10%), *iss* (6.5%), and iucA (5.2%; Figure 4).

### 3.6. Plasmid Typing

A total of nine plasmid replicons were identified in all metagenomic samples. Plasmid replicons were identified as Inc (IncFIA, IncFIB, IncN), rep (*repUS12*, rep7), and Col (ColRNAI) types. Type IncF was found to be the major replicon type (69.2%), followed by rep (23%) and Col (7.8%) types (Table 2).

## 4. Discussion

Before the advent of NGS, conventional bacterial culture methods were mostly used for AMR studies [30]. However, the emergence of NGS shifted the trend from studying a single culture microorganism to the level of microbial communities [31]. A cross-sectional metagenomic study was designed to evaluate the fecal microbiome and its resistome in broiler chickens infected with MDR *E. coli*.

In Pakistan, antimicrobials are excessively administered in poultry for both therapeutic and non-therapeutic purposes [10], which may have led to the emergence of various ARG types in the gut microbiota of humans and animals. In the present study, multidrug efflux pump-associated ARGs were found to be the most abundant genes. ARGs conferring resistance to aminoglycoside, tetracycline, MLS, and beta-lactam were the other prevalent genes. The abundance of ARGs conferring resistance to multidrug, aminoglycoside, and tetracycline is in agreement with a prior broiler chicken metagenomic report [12]. The common types of various ARGs identified in the present study have been reported in fecal samples derived from human, poultry, and environmental sources [32,33,34,35].

The presence of common ARG types can be associated with the pattern of antibiotics administration at a particular poultry farm. A previous study associated the prevalence of various ARG types (β-lactams, aminoglycosides, and tetracycline) to the common administration of these antibiotics in the respective poultry farms [35]. Data related to antibiotic usage (AMU) in poultry farms of Pakistan are unregulated; however, commonly used antibiotics for growth promotion in poultry farms are mainly β-lactams, lincosamides, tetracyclines, and macrolides [10,36]. The prevalence of various ARG types in the present study could be due to the selective pressure imposed by these particular antibiotics administered in broiler chickens. Linking the presence of specific ARGs to particular sources has been considered difficult; however, the various feed and environmental sources influence the acquisition of particular ARG types in the gut resistome [35]. The high prevalence and diversity ARGs in our study might be due to the excessive use of antibiotics and the absence of AMU regulation in Pakistan’s poultry. Our suspicion of linking the excessive use of antibiotics to the high prevalence of ARGs is supported by a previous report [37]. Moreover, a Chinese research group constructed a poultry gut microbiome catalog and compared the diversity and abundance of various ARG types with gene sets of pig and human. Their findings revealed that the abundance of various ARGs was high in poultry compared to the pig and human. The high prevalence of various ARG types in poultry was linked to the excessive use of various antimicrobials in poultry feed [37].

Plasmids are considered an important source for the transmission of various ARGs between different species through horizontal gene transfer [38]. Resistome analysis identified various plasmids’ replicon types, namely IncF, Col, and rep, in all the samples. IncF was identified as the major plasmid replicon type, which agrees with the previous studies indicating that IncF is a common plasmid replicon type in various avian species of Pakistan [11,19,20].

Bacteria invade and overcome the host defense system using virulence factors. The predominant virulence-associated genes (VAGs) identified were *iro*N, *iut*A, *iss*, and *iuc*A. The VAGs identified in our study are different from the previous studies (11, 20). Using multiplex polymerase chain reaction (PCR), Azam et al. [20] reported that the most abundant VAGs in avian pathogenic *E. coli* infected broilers (APEC) are iss (84%), followed by *iut*A (74.6%), *Col*V (60%), *tsh* (57.3%), and *iro*N (57.3%). Similarly, another study, in opposition to our study, reported that the most prevalent VAGs in *E. coli* infected poultry were *iss* (78.2%), followed by *iro*N (60.9%), and *lpf*A (59.8%) [11]. The difference in the prevalence of VAGs identified in our study to those of previous studies can be attributed to the different sampling regions and the use of different molecular methods. The prevalence pattern of various VAGs identified in the present study is supported by an Egyptian study indicating the prevalence of *iro*N, *iss*, *iut*A in MDR APEC infected broilers [39].

Firmicutes have been reported to be the major phylogenetic group in poultry, while Bacteroidetes and Proteobacteria are the other dominant phyla in chickens [40,41,42]. Distributions of bacterial phyla in our study are different from the previous studies. Several factors change the microbial composition of the gut including various environmental factors, antibiotics, age, and diet [43]. Among the various factors, antibiotics have been reported to cause substantial changes to the gut microbial composition. The effect of various antimicrobials on the gut microbial composition of chicks has been explored by a recent study. The gut microbial composition of chicks was found to be altered upon exposure to antibiotics such as diclazuril, enrofloxacin, and their combinations. Exposure to various antibiotics decreased the predominance of phyla Actinobacteria, Firmicutes, Thermi, and Verrucomicrobia [43]. Our results showed the predominance of phylum Proteobacteria followed by Firmicutes, Bacteroidetes, and Tenericutes. The high abundance of Proteobacteria is in agreement with a previous study [35]. The possible contributing factor for the abundance of Proteobacteria can be excessive antibiotic administration in poultry feed, which modified the microbial community structure of broiler chickens. Furthermore, a high abundance of Proteobacteria changes the gut microbial composition, thereby leading to microbial dysbiosis [44]. The beta-oxidation ability of epithelial cells reduces during an intestinal inflammation, thereby leading to an increased availability of oxygen, which in turn possibly causes the expansion of Proteobacteria and microbial dysbiosis [44,45].

At the family level, we observed an increased abundance of Enterobacteriaceae in the present study. Blooms of Enterobacteriaceae have been associated with an inflamed gut microenvironment [46]. During inflammatory conditions, the host cell produces nitrate, which is thought to be exploited by the blooms of Enterobacteriaceae [47]. Therefore, the predominance of Proteobacteria and Enterobacteriaceae in the present study indicates intestinal inflammation, leading to microbial dysbiosis. The microbial dysbiosis in broilers can be linked to antibiotic resistance. Moreover, the association of microbial dysbiosis with antibiotic resistance in poultry could be supported by a previous study linking the predominance of Proteobacteria with microbial dysbiosis in ilea of nephropathogenic infectious bronchitis virus-infected chickens [48]. The over dominance of various pathobionts (*E. coli*, *Klebsiella pneumonia*, *Salmonella enterica*, *Shigella flexneri*) in this study is supported by the fact that an increased abundance of Proteobacteria has been linked with the emergence of pathogenic microbes [49,50]. Furthermore, the abundance of *E. coli* in the current study is supported by a prior chicken metagenomic study [35]. *E. coli* is considered an important gut pathobiont and its abundance can pose a serious threat to human and food animals.

## 5. Conclusions

The present study documents the widespread presence of diverse ARGs, plasmid replicon types, VAGs, and dysbiotic (modified) microbiota in broiler chickens of Khyber Pakhtunkhwa infected with MDR *E. coli*. In addition to the widespread presence of ARGs, plasmid replicon types and VAGs, our study indicated the possible associations of poultry gut microbial dysbiosis with antimicrobial resistance. The gut microbial profiling of MDR *E. coli* infected broiler chickens can help in designing strategies for targeted mitigation of resistant microbes. Furthermore, this study highlighted the use of SSMS as a suitable, robust, high throughput, and cost-effective sequencing approach for both the resistome and fecal microbiome profiling.

## Figures and Tables

**Table 1:** Types of antibiotics genes identified using next-generation sequencing in the resistome of multidrug-resistant *E. coli* infected broilers.

Resistant Drug Class	Antibiotic-Resistant Genes
**Multidrug efflux pumps**	*cfr*C, *acr*E, *Mex*F, *acr*D, *arn*A, *sat*4, *Mex*K, *acr*A, *acr*B, *bac*A, *bae*R, *bae*S, *CRP*, *evg*A, *evg*S, *ept*A, *gad*W, *gad*X, *pmr*F, *ugd*, *Mex*D, *Mex*M, *Mex*Q, *mef*B, *mux*C, *Opr*M, *Opm*D, *Mex*X, *bas*S, *kdp*E, *mdt*A, *mdt*B, *mdt*C, *mdt*E, *mdt*F, *mdt*G, *mdt*H, *mdt*K, *mdt*M, *mdt*N, *mdf*A, *msb*A, *mar*A, *mex*Y, *Omp*K37, *tol*C, *yoj*I, *vga*C, *sme*E, *sme*R, *Mex*A, *Mex*B, *Mex*C, *Mex*E, *Mex*H, *Mex*I, *Mex*J, *Mex*L, *Mex*N, *Mex*V, *Mex*W, *Mux*A, *Mux*B, *Opm*B, *Opr*J, *acr*S, *Opr*N, *sox*R, *bcr-1*, *spd*
**Aminoglycoside**	*APH*3-III, *APH*3-IIIa, *Sat*4A, *aad*E, *aad*S, *aad*9, *ant*6-Ia, *aad*6, *APH*7, *ant6*-Ib, *aac*6-*aph*2, *aac*6-Ie-aph2, *aad*A12, *Str*B, *APH*(6)-Id, *StrA*, *aac*6-Im, *aad*C,*aad*D, *APH*-Stph
**Tetracycline**	*Tet*W,*Tet*32, *Tet*A, *Tet*Q, *Tet*(W/N/W), *Tet*44, *Tet*40, *Tet*B, *Tet*C, *Tet*O, *Tet*X, *Tet*L, *Tet*34, *Tet*M, *Tet*R
**MLS** ^1^	*emr*B, *emr*B_18, *Lsa*B, *emr*A, *emr*G, *emr*K, *emr*Y, *Mef*B, *Lnu*C, *emr*B_16, *emr*C, *emr*F, *emr*R, *Msr*A, *mef*(En2), *lnu*C, *Iuc*D, *lsa*E, *lnu*(AN2)
**Beta-lactam**	*blaAmp*H, *blaAmp*C2, *blaTEM-*1, *blaTEM-*102, *blaTEM-*104, *blaTEM-*105, *blaTEM-*112, *blaTEM-*116, *blaCTX-*M-101, *CTX-*M-107, *blaAZECL-*25, *blaNDM-*1, *blaNDM-*11, *blaCfx*A, *blaCfx*A2, *blaCfx*A6, *blaOXY-*2, *blaOXA-*1, *MIR-*13, *blaCMY-*111, *blaCMY-*17, *blaCTX-*M-144, *blaCTX-*M-144, *blaCTX-*M-52, *blaOXA-*181, *blaCMH-*2, *blaCMH-*3
**Rifampin**	*rpo*B, *rpo*B2
**Sulphonamide**	*sul*, *sul*2, *sul*3,
**Phenicol**	*cat*B4, *flo*R
**Vancomycin**	*van*R
**Trimethoprim**	*Dfr*G
**Colistin**	*mcr-*1
**Quinolone**	*Oqx*AB

^1^ MLS = macrolide-lincosamide-streptogramin.

**Table 2:** List of prevalent plasmid replicon types identified in all the poultry metagenomic samples.

Plasmid Replicon Types	Accession Number
IncFIA	AP001918
IncFIB	AP001918
IncFII	AY458016
IncFIC	AP001918
IncFII_1_pSFO	AF401292
IncN_1	AY046276
repUS12._rep_pUB110	AF181950
rep7.17_repC_pS0385	AM990993.1
ColRNAI_1	DQ298019

## Supplementary Materials

The following are available online at https://www.mdpi.com/2076-2615/10/12/2350/s1, Table S1: Spectrum of antibiotics used in the selected poultry farms for therapeutic and growth promotion purposes, Table S2: Screening of *E. coli* infected samples among various poultry flocks.

## Availability of Raw Sequencing Data

Raw metagenomic data generated in this study has been deposited to the publicly accessible NCBI Sequence Read Archive (SRA) under the accession number: PRJNA612780.
