# Supplementary material for: Next-Generation Sequencing Based Gut Resistome Profiling of Broiler Chickens Infected with Multidrug-Resistant Escherichia coli
Source: Animals (Basel). 2020 Dec 9;10(12):2350. doi: 10.3390/ani10122350 (PMC7764233; doi:10.3390/ani10122350)
Supplement: Supplementary file 1 [file animals-10-02350-s001.pdf]

Table S1. Spectrum of antibiotics used in the selected poultry farms for therapeutic and growth promotion purposes.

Antibiotics	Class	Purpose of Use
Tylosin	Macrolide	Therapeutic
Doxycycline	Tetracycline	Therapeutic
Amoxicillin	Aminopenicillins	Therapeutic
Colistin	Cyclopolypeptides	Therapeutic and growth promotion
Norfloxacin	Fluoroquinolone	Therapeutic mainly
Enrofloxacin	Fluoroquinolone	Therapeutic mainly
Lincomysin	Lincosamide	Growth promotion
Virginiamycin	Streptogramins	Growth promotion
Neomycin	Aminoglycoside	Growth promotion
Tetracycline	Tetracycline	Therapeutic and growth promotion
Spiramycin	Macrolide	Therapeutic
Bambermycin	Phosphoglycolipids	Growth promotion
Sulfonamides	Sulfonamides	Therapeutic and growth promotion
Streptomycin	Aminoglycoside	Therapeutic
Bacitracin	Cyclic peptide	Therapeutic and growth promotion
Erythromycin	Macrolides	Therapeutic
Monensin	Ionophores	Growth promotion and for the treatment of coccidiosis

Table S2. Screening of E. coli infected samples among various poultry flocks.

Poultry Farms	*E. coli* Positive Samples (%)
Chargano Chowk Peshawar	30(12.4)
Chamkani, Peshawar	17(7)
Dabgari Garden, Peshawar	15(6.2)
Mardan	16(6.6)
Dalazak road, Peshawar	18(7.4)
University town, Peshawar	18(7.4)
Charsadda	23(9.5)
Malakand	24(9.9)
Mohmand Agency	15(6.2)
Swabi	18(7.4)
Kohat Road, Peshawar	15(6.2)
Nowshera	20(8.3)
Hayatabad, Peshawar	13(5.4)
